# Expression of chemokine receptors on circulating tumor cells in patients with solid tumors

**DOI:** 10.1186/1479-5876-10-52

**Published:** 2012-03-20

**Authors:** Alberto Fusi, Zhian Liu, Verena Kümmerlen, Anika Nonnemacher, Judith Jeske, Ulrich Keilholz

**Affiliations:** 1 Department of Hematology and Medical Oncology, Charité, Campus Benjamin Franklin, Hindenburgdamm 30, 12200 Berlin, Germany

**Keywords:** Circulating tumor cells, Chemokine receptor, CD45 depletion, CXCR4, CCR6, CCR7, CCR9

## Abstract

**Background:**

The study was performed to investigate the expression of chemokine receptors (CR) on circulating tumor cells (CTC), which may be of importance for organ-specific metastases and cancer treatment since CR are potential drug-targets.

**Methods:**

Blood samples from patients with metastatic carcinoma (MC) or melanoma (MM) were enriched for CTC and expression of CR (CXCR4, CCR6, CCR7 and CCR9) was evaluated by flow cytometry.

**Results:**

CTC were detected in 49 of 68 patients (72%) [28 MC; 21 MM] with a median number of 3 CTC (range: 1-94)/10 mL of blood. CXCR4 was expressed on CTC in 82% (40/49) of patients [median number 1 CTC/10 mL blood; range 1-14] and CCR6 in 29 patients (59%; median 1, range: 1-14). In MM patients, CCR7 was expressed on CTC in 6 (29%) samples and CCR9 in 12 (57%). A positive correlation between surface expression of CR and organ-specific metastatic pattern was not observed.

**Conclusions:**

CR were expressed on CTC of patients with solid tumors. Along with our findings, the observation that CR could be involved in CTC proliferation and migration of tumor cells appoints CTC as potential CR-antagonist therapeutic target.

## Background

The presence of circulating tumor cells (CTC) was first described in 1869 by Thomas Ashworth. Cells similar to the ones of the tumor were observed in the blood of a man with metastatic cancer [1]. Despite improvements in isolation and in characterization of CTC, the current understanding of their biological properties is very limited. It is in fact totally unclear, whether CTC are a fraction of cells transiently present in the blood stream as a prerequisite to potentially seed haematogenous metastases of the disease, or represent a unique subpopulation of tumor cells able to survive and circulate in the blood stream for an extended duration, perhaps with the potential to eventually home to peripheral tissues, where they may or may not be able to initiate formation of metastases (i.e. possess complete metastasis-initiating properties).

Presence of tumor cells in the circulation does not necessary end up with development of metastasis and its growth could be a phenomenon due to random survival of few tumor cells. In a mouse model it has indeed been shown that less than 0.1% of tumor cells of the primary tumor survived in blood to produce metastases [2]. However, although CTC might be simply shed in the circulation without having a clinical impact, a significant correlation between the presence of CTC and development of distant metastases and outcome has been observed by several groups [3-7].

Metastases show in the majority of cases an organ-specific pattern of spread and this specificity is independent from any anatomical factor. In 1889 Stephen Paget analyzed 735 autopsy records of women with breast cancer and a non-random pattern of organ metastases was observed. Paget's results led to the formulation of the so called "seed and soil theory" [8]. The process of metastasis seemed therefore not due to chance, but to the fact that tumor cells (the 'seed') had a specific affinity for the microenvironment of certain organs (the 'soil'). Different organs have special abilities to arrest, attract, and promote certain types of cancer cells which matched the specific microenvironment of the host tissue. A major mechanism in the seed concept is that metastatic cancer cells co-opt chemokine-mediated signalling (seed-factor), which normally controls leukocyte distribution.

Chemokines display pleiotropic effects in immunity, regulating angiogenesis, promoting proliferation of tumor cells and mediating organ-specific metastases [9]. Several different chemokine receptors (CR), including CXCR4 [10-15], CCR7 [16-18], CCR9 [19] and CCR6 [20,21], have been suggested to mediate metastasis to specific target-organs and the presence of a specific CR on cancer cells has been associated with a definite metastatic pattern.

In particular, high levels of functional CXCR4 receptors have been observed on human breast cancer cells and correspondingly, the highest CXCR4 ligand expression, CXCL12, was detected in organs that are preferential destinations of breast cancer metastasis [10]. Other studies supported the idea that the CXCR4/CXCL12 axis is the principle mechanism for marrow homing of normal or malignant cells and may therefore regulate migration and metastasis of a variety of cancer types including melanoma and colon cancer [11-15]. Expression of CCR7 in tumor cells of patients with non-small-cell lung cancer or colon cancer has been correlated with the ability of the cells to spread to the lymph nodes [16,17]. In a previous study we observed a strong correlation between functional CCR9 expression in melanoma and the occurrence of intestinal metastases [19]. Similarly, we observed a striking correlation between CCR6 overexpression and synchronous liver metastasis in patients with colorectal carcinoma [20].

In this study we conducted an exploratory analysis to evaluate whether CTC enriched from patients with solid tumors expressed the CR CXCR4, CCR6, CCR7 and CCR9. Correlation between expression of CR and metastatic pattern was also evaluated.

## Methods

### Samples collection

The investigation was approved by the Ethic Committee at Charité. Sixty-eight consecutive patients receiving various forms of systemic chemotherapy at Charité (Berlin, Germany) were enrolled: 29 were affected by metastatic melanoma [MM] and 39 by metastatic carcinoma [MC]. Twenty mL blood anticoagulated with heparin was collected after informed consent from each patient. Blood was collected before treatment start irrespective of the line of treatment. Samples were drawn after discarding the first 2 mL of blood to avoid potential skin cell contamination from venipuncture and processed within 1 hour after sampling.

### Enrichment (CD45 depletion) for CTC

Red blood cell lysis buffer (154 mM NH4Cl, 10 mM KHCO_3 _and 0.1 mM EDTA in deionized water) was used to lyse erythrocytes. Cells were subsequently washed with a buffer consisting of phosphate-buffered saline (PBS) containing 0.5% bovin serum albumin (BSA), and 2 mM ethylenediaminetetraacetic acid (EDTA). Cells were counted and resuspended in the buffer at a concentration of 1 × 10^8 ^cells/mL and then enriched for tumor cells by CD45 depletion of the leukocyte fraction using a magnetic bead separation technique (EasySep^®^, Stem Cells Technologies, Inc., Vancouver, BC, Canada) as previously described [22]. The remaining material was split in two fractions and stained with either a cocktail of specific antibodies or with the corresponding isotypic control antibodies purchased from the same manufacturer. All antibody batches were titrated to determine their optimal concentration. Specificity, recovery and linearity of the method have been previously reported [22-24].

### Characterization of CTC for CR expression by flow cytometry

In case of carcinomas cells were stained on the surface with a cocktail containing EpCAM (0.006 mg/mL; clone EBA-1, BD Biosciences, San José, CA, USA), CXCR4 (0.002 mg/mL; clone 12 G5; BD Biosciences); CCR6 (0.002 mg/mL; clone R6H1, eBiosciences San Diego, CA, USA) and CD45 (0.015 mg/mL clone TU116, BD Biosciences). For intracellular staining cells were fixed with 1% formaldehyde and thereafter permeabilized. Briefly, pellet was resuspended in 2 mL of a sterile solution containing 0.1% saponin, 0.05% NaN_3 _in Hanks' Balanced Salt Solution (SAP buffer). Cells were centrifuged at 200 × g for 5 minutes; supernatant decanted ensuring that approximately 200 μL of SAP buffer remained in the tube. Cells were subsequently stained with antibodies specific for cytokeratin (CK) 7 and 8 (0.01 mg/mL clone CAM 5.2, BD Biosciences) and incubated for 20 minutes in the dark at 4°C.

In case of melanoma, cells were stained on the surface with melanoma-associated chondroitin sulphate proteoglycan (Miltenyi Biotec Inc., Auburn CA, USA, CD45 (clone TU116, BD Biosciences), CXCR4 (BD Biosciences); CCR6 (eBiosciences); CCR7 (0.002 mg/mL; Clone 150503, R&D System, Minneapolis, MN USA) and CCR9 (0.002 mg/mL; clone 112509, R&D System).

Cells were acquired on a FACSCanto II system (BD Biosciences) and the whole volume was evaluated. Potential epithelial cells were defined as EpCAM and CK double-positive and CD45 negative. Potential melanoma cells were defined as melanoma-associated chondroitin sulphate proteoglycan-positive and CD45 negative. Data were analysed with the use of FlowJo 7.2.5 software (Tree Star, Ashland, OR, USA).

### Statistical analysis

Chi-squared or Fisher's exact test tests were used where appropriate to evaluate any differences between the parameters examined. Two-sided p < 0.05 was considered to be statistically significant. The analysis was performed using SPSS Inc. Software (version 18.0).

## Results

### Patients and detection of CTC

CTC were detected in the blood of 49 patients (72%): 28 out of 39 (72%) MC patients and 21 out of 29 (72%) MM patients showed at least 1 CTC/10 mL blood. Characteristics of the patients included in the analysis on CR expression (positive for presence of CTC) are listed in Table 1.

**Table 1:** Clinical characteristics of the patients included in the analysis

	Total (n = 49)	MC (n = 28)	MM (n = 21)
Age, median (range)	63 (25-85)		

Gender (M:F)	25:24		

Primary tumor			
Colon cancer		9	
Breast cancer		5	
NSCLC		4	
Cervix cancer		3	
Ovarian cancer		3	
SCCHN		2	
Pancreatic cancer		2	
Cutaneous Melanoma			11
Uveal melanoma			10

Number of metastatic sites			
1	27		
2	15		
> 2	7		

Liver metastasis			
Yes	26	11	15
No	23	17	6

Lung metastasis			
Yes	22	16	6
No	27	12	15

MC: Metastatic carcinoma; MM: metastatic melanoma; NSCLC: non-small-cell lung cancer; SCCHN: Head and neck squamous cell carcinoma

The median number of CTC was 3 (range: 1-94)/10 mL. The median number of CTC was 2 (range: 1-21)/10 mL blood in the MC cohort and 4 (range: 1-94)/10 mL blood in the MM group.

### Expression of chemokine receptors on CTC

**Figure 1:**
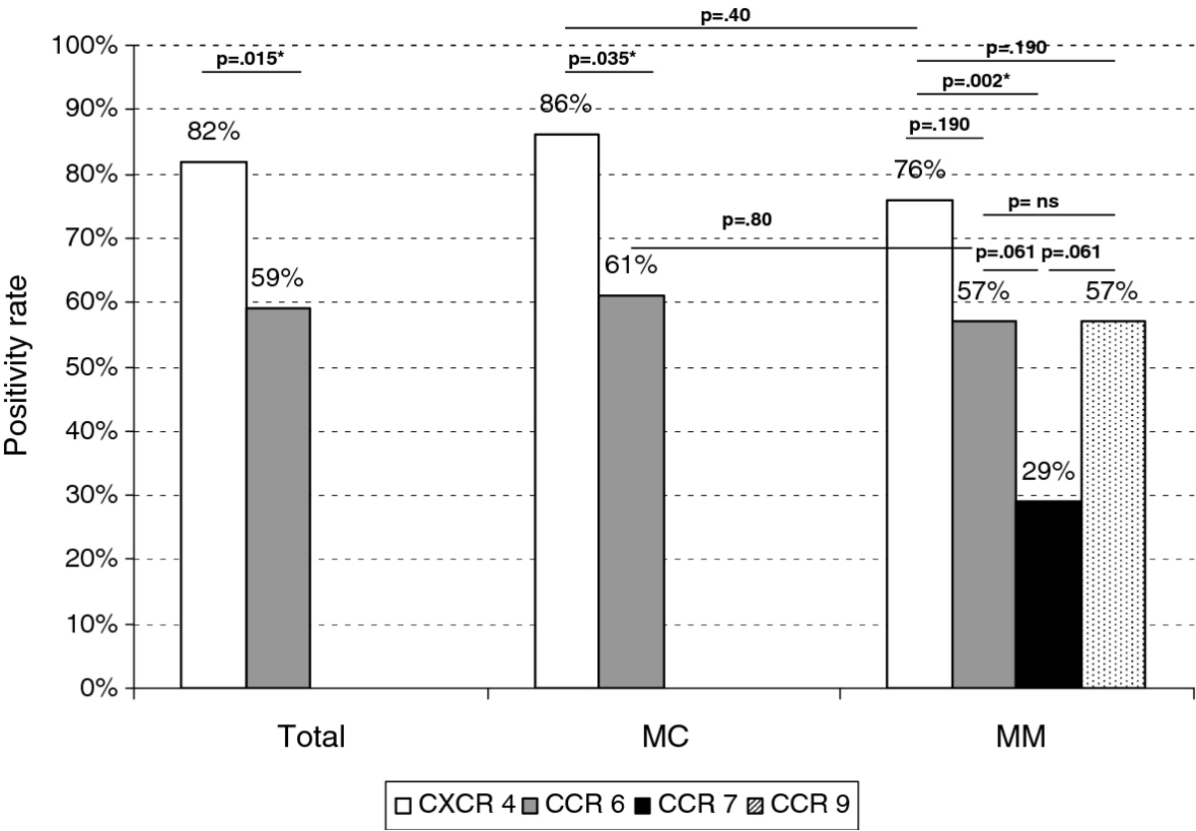
**Expression positivity rates (number of samples in which CR-expressing CTC were observed/number of CTC-positive samples evaluated) of the chemokine receptors CXCR4, CCR6, CCR7, and CCR9 on CTC in the whole population or in patients with metastatic carcinoma (MC) or metastatic melanoma (MM)**. Significant differences are indicated by an asterisk (*).

Results were summarized in Table 2 and depicted in Figure 1. CXCR4 was expressed on CTC in 82% (40/49) of the patients and the median number of CXCR4-positive CTC was 1 (range: 1-14). CCR6 was expressed in 29 patients (59%) and the median number of CCR6positive CTC was 1 (range: 1-14). Expression rates of CXCR4 and CCR6 did not significantly differ between MC and MM patients (Figure 1). CXCR4 was more frequently expressed on CTC than CCR6 (p = 0.015; Figure 1).

**Table 2:** Median number of chemokine receptor-positive cells and positivity rates (PR)

		Total (n = 49)	MC (n = 28)	MM (n = 21)
CXCR4	PR Median [range]	82% (40/49) 1 [1-14]	86% (24/28) 1 [1-14]	76% (16/21) 2 [1-8]

CCR6	PR Median [range]	59% (29/49) 1 [1-14]	61% (17/28) 1 [1-14]	57% (12/21) 2 [1-8]

CCR7	PR Median [range]			29% (6/21) 1 [1-5]

CCR9	PR Median [range]			57% (12/21) 2 [1-5]

MC: Metastatic carcinoma; MM: metastatic melanoma

CCR7 was expressed on CTC in 6 MM patients (6/21 = 29%) and the median number of CCR7-positive CTC was 1 (range: 1-5). CCR9 was expressed on CTC in 12 MM patients (57%) and the median number of CCR7-positive CTC was 2 (range: 1-5).

Seventeen (61%) patients with MC presented CTC double positive for CXCR4 and CCR6; 7 (25%) patients showed CTC positive for CXCR4 or CCR6 and 4 (14%) had CTC negative for both the CXCR4 and CCR6. The median number of CTC without expression of CXCR4 and CCR6 was 1 (range: 1-18). Fourteen (66%) patients affected by MM presented CTC positive for at least two of the CR analysed; 3 (14%) patients showed CTC positive for only one CR and 4 (19%) resulted to have CTC negative for expression of all the CR evaluated. The median number of CTC negative for all the CR analysed was 6 (range 3-86).

### Expression of chemokine receptors on CTC and metastatic pattern

We then asked if expression of the CR on CTC correlated with presence of liver metastasis or lung metastasis. Results are presented in Table 3. No positive correlation was observed between expression of CR on CTC and presence of liver or lung metastasis. Results did not change when MM and MC patients were analysed separately and when analyses were performed including only patients with solely liver metastases (n = 15) vs. patients without liver metastasis (data not shown). Presence of liver or lung metastasis was not associated with the concurrently expression of two different CR (data not shown).

**Table 3:** Correlation between chemokine receptor expression and presence of liver or lung metastases

	Liver mestastasis			Lung metastasis		
				
	Yes (%)	No (%)	p	Yes (%)	No (%)	p
CXCR4	21/26 (81%)	19/23 (83%)	0.28	17/22 (77%)	23/27 (85%)	0.44
CCR6	14/26 (54%)	15/23 (65%)	0.42	13/22 (59%)	16/27 (62%)	0.84
CCR7 *	5/15 (33%)	1/6 (17%)	0.42	0/6 (0%)	6/15 (40%)	0.09
CCR9 *	9/15 (60%)	3/6 (50%)	0.34	1/6 (17%)	11/15 (73%)	0.03

* only patients with metastatic melanoma

## Discussion

In the present study, we investigated a series of blood samples drawn from consecutive metastatic patients with different solid tumors, in order to evaluate the expression of the CR CXCR4, CCR6, CCR7 and CCR9 on CTC. CTC expressed CR in the majority of cases, with CXCR4 being the most frequently expressed CR and CCR6 often co-expressed.

The studies published so far about this topic are not directly comparable with our study due to profound differences in strategies employed for identification of CTC. These reports, although more homogeneous in terms of patients, did not enrich the blood specimens for CTC and they used a more extensive definition for CTC identification. Pituch-Noworolska et al. evaluated the immunophenotype of CK+PBMCs and of disseminated tumor cells (DTC) from bone marrow in patients with gastric cancer. CCR6 and CXCR4 expression was determined on CK+cells sorted out from blood (8 samples) or bone marrow (69 samples). CCR6 was expressed in a half of the CK+samples whereas the expression of CXCR4 was much lower [25]. We also observed expression of CCR6 in a half of our samples, but unlike Pituch-Noworolska we observed a much higher expression of CXCR4. High levels of CXCR4 expression (> 90%) on CK+PBMCs were also observed by other authors in small pilot studies involving patients with non-small cell lung cancer [26] or patients with metastatic renal cancer [27]. For definition of CTC, these studies used only CK as epithelial marker. Although there is no reference standard, the definition of epithelial CTC as EpCAM +CK+CD45- we utilized, is the wider employed and the most accepted. This is indeed the same definition for identification of CTC applied by the Cell Search System which was cleared by the FDA for detection of CTC in some cancer entities. Albeit we agree that the actual definition might be too restrictive [22], it has been reported that there are 0-20% CK+ cells in blood samples from normal adults which might increase the rate of false positive samples and invalidate the analysis. As first exploratory investigation, we therefore decided not to focus on a single cancer type; on the contrary we preferred to evaluate a broader range of cancer and a larger spectrum of chemokine receptors.

Despite previous observations of an association between expression of specific CR in primary tumors and metastatic organ preference, we did not find any correlation between CR expression on CTC and specific metastatic pattern. In our study we evaluated the presence of CR on the surface of CTC (i.e. ready-to-home cells) without analysing their intracellular expression. Intracellular expression of CR (in particular nuclear expression of CXCR4) assessed at primary tissue level has been associated to the metastatic destination of tumor cells and to patient outcome in several different cancer entities [16-20,28-30]. It might be therefore possible that we underestimated the quote of CR-positive CTC. This implies that a further step is needed before specific cell-seeding: CR already present in the cell should migrate to the surface. Migration could be trigged by several mechanisms including unspecific adhesion [31] and hypoxia [32,33]. Another limitation of our study consisted in the cohort of patients we analysed. Blood samples were in fact drawn from patients with distant metastases affected by different solid tumors in a relative small number of cases. A substantial conclusion could not therefore be drawn. An eventual correlation between CR profile on CTC and metastatic pattern could have been better assessed in a cohort of patients with the same tumor entity and before development of distant metastasis. The biology of the CTC in patients with metastasis is poorly investigated, but recent data suggested they might be a source of further tumor foci. Massagué and colleagues showed that CTC from distant metastasis were able to re-infiltrate tumors at their organs of origin and even to promote the growth of the primary tumors [34]. However, studies which evaluate expression of more than one CR in patients with metastatic tumors both at primary and metastatic level demonstrated a more varied CR expression at primary level [35-37] compared to metastases and similar to the expression profile on CTC we observed. That might denote that the seed (CTC) is 'equipped' to migrate potentially anywhere and that the soil has the principal role in directing migration of the seed to specific sites. This statement is consistent with the fact that conditioned media obtained from distinct tumor types with unique patterns of metastatic spread redirected premetastatic niche formation, thereby transforming the metastatic profile, in Id3 knockout mice [38].

Presence of CR on CTC makes CTC a potential therapeutic target for CR antagonists. The recent approval of a CCR5 receptor antagonist in HIV paves in fact the way for further effective antagonists to other CR. Various strategies have been employed in preclinical models to target CXCR4 including inhibitory antibodies, small molecule antagonists, RNAi and small inhibitory peptides. CXCR4-directed antibodies suppressed lymph node metastasis in experimental breast cancer [10] and suppressed tumor growth and impaired the development of tumor endothelium in experimental models of colon and pancreatic cancer [39]. Administration of the specific small molecule CXCR4 antagonist AMD3100 to mice with intracranial glioblastoma effectively inhibited growth and increased apoptosis of the tumor cells [40]. Similarly, AMD3100 inhibited ascites accumulation in an experimental model of gastric cancer [41]. CTCE-9908 is a peptide analogue of SDF-1 that acts as a competitive antagonist of CXCR4. In experimental models, treatment with CTCE-9908 showed to reduce tumor burden, but did not reduce the frequency of metastasis suggesting that other mechanisms independent from the expression of CXCR4 are involved in metastasis development [42,43]. Although investigation of the role of CR inhibitors in clinical setting is still in the earlier phase [44], presence of CXCR4 at high frequency on CTC of patients with solid tumors makes CTC a potential therapeutic target for CXCR4 antagonists in a large number of patients. However, functional assays evaluating the role of CR in migration and proliferation of CTC are necessary to assess if a CR inhibition strategy could be taken into consideration.

## Conclusions

CR are expressed on CTC of patients with metastatic solid tumors. Even though in this study we did not find a positive correlation between CR expression on CTC and metastatic pattern, these receptors could be involved in CTC proliferation and migration of cancer cells, which appoints CTC as potential CR-antagonist therapeutic targets.

## Abbreviations

CTC: Circulating tumor cells; CR: Chemokine receptors; MC: Metastatic carcinoma; MM: Metastatic melanoma.

## Competing interests

The authors declare that they have no competing interests.

## Authors' contributions

AF participated in the design of the study and drafted the manuscript. ZL, AN and JJ carried out the experiments. VK participated in sample collection. UK participated in the design of the study and sample collection and drafted the manuscript. All authors read and approved the final manuscript.

## References

[B1] Ashworth TR A case of cancer in which cells similar to those in the tumors were seen in the blood after death Australian Medical Journal 1869 14 146 147

[B2] Fidler IJ Metastasis: quantitative analysis of distribution and fate of tumor emboli labeled with 125I-5-iodo-2'-deoxyuridine J Natl Cancer Inst 1970 45 773 782 5513503

[B3] Cohen SJ Punt CJ Iannotti N Saidman BH Sabbath KD Gabrail NY Picus J Morse M Mitchell E Miller MC Doyle GV Tissing H Relationship of circulating tumor cells to tumor response, progression-free survival, and overall survival in patients with metastatic colorectal cancer J Clin Oncol 2008 26 3213 3221 10.1200/JCO.2007.15.8923 18591556

[B4] Danila DC Heller G Gignac GA Gonzalez-Espinoza R Anand A Tanaka E Lilja H Schwartz L Larson S Fleisher M Scher HI Circulating tumor cell number and prognosis in progressive castration-resistant prostate cancer Clin Cancer Res 2007 13 7053 7058 10.1158/1078-0432.CCR-07-1506 18056182

[B5] Hiraiwa K Takeuchi H Hasegawa H Saikawa Y Suda K Ando T Kumagai K Irino T Yoshikawa T Matsuda S Kitajima M Kitagawa Y Clinical significance of circulating tumor cells in blood from patients with gastrointestinal cancers Ann Surg Oncol 2008 5 3092 3100 18766405 10.1245/s10434-008-0122-9

[B6] Pachmann K Camara O Kavallaris A Krauspe S Malarski N Gajda M Kroll T Jörke C Hammer U Altendorf-Hofmann A Rabenstein C Pachmann U Monitoring the response of circulating epithelial tumor cells to adjuvant chemotherapy in breast cancer allows detection of patients at risk of early relapse J Clin Oncol 2008 26 1208 1215 10.1200/JCO.2007.13.6523 18323545

[B7] Riethdorf S Fritsche H Müller V Rau T Schindlbeck C Rack B Janni W Coith C Beck K Jänicke F Jackson S Gornet T Detection of circulating tumor cells in peripheral blood of patients with metastatic breast cancer: a validation study of the Cell Search system Clin Cancer Res 2007 13 920 928 10.1158/1078-0432.CCR-06-1695 17289886

[B8] Paget S The distribution of secondary growths in cancer of the breast Lancet 1889 1 571 573 2673568

[B9] Charo IF Ransohoff RM The many roles of chemokines and chemokine receptors in inflammation N Engl J Med 2006 354 610 621 10.1056/NEJMra052723 16467548

[B10] Müller A Homey B Soto H Ge N Catron D Buchanan ME McClanahan T Murphy E Yuan W Wagner SN Barrera JL Mohar A Involvement of chemokine receptors in breast cancer metastasis Nature 2001 410 50 56 10.1038/35065016 11242036

[B11] Li YM Pan Y Wei Y Cheng X Zhou BP Tan M Zhou X Xia W Hortobagyi GN Yu D Hung MC Upregulation of CXCR4 is essential for HER2-mediated tumor metastasis Cancer Cell 2004 6 459 469 10.1016/j.ccr.2004.09.027 15542430

[B12] Kaifi JT Yekebas EF Schurr P Obonyo D Wachowiak R Busch P Heinecke A Pantel K Izbicki JR Tumor-cell homing to lymph nodes and bone marrow and CXCR4 expression in esophageal cancer J Natl Cancer Inst 2005 97 1840 1847 10.1093/jnci/dji431 16368946

[B13] Darash-Yahana M Pikarsky E Abramovitch R Zeira E Pal B Karplus R Beider K Avniel S Kasem S Galun E Peled A Role of high expression levels of CXCR4 in tumor growth, vascularization, and metastasis FASEB J 2004 18 1240 1242 15180966 10.1096/fj.03-0935fje

[B14] Marchesi F Monti P Leone BE Zerbi A Vecchi A Piemonti L Mantovani A Allavena P Increased survival, proliferation, and migration in metastatic human pancreatic tumor cells expressing functional CXCR4 Cancer Res 2004 64 8420 8427 10.1158/0008-5472.CAN-04-1343 15548713

[B15] Zeelenberg IS Ruuls-Van Stalle L Roos E The chemokine receptor CXCR4 is required for outgrowth of colon carcinoma micrometastases Cancer Res 2003 63 3833 3839 12839981

[B16] Takanami I Overexpression of CCR7 mRNA in non small cell lung cancer: correlation with lymph node metastasis Int J Cancer 2003 105 186 189 10.1002/ijc.11063 12673677

[B17] Günther K Leier J Henning G Dimmler A Weissbach R Hohenberger W Förster R Prediction of lymph node metastasis in colorectal carcinoma by expression of chemokine receptor CCR7 Int J Cancer 2005 116 726 733 10.1002/ijc.21123 15828050

[B18] Wang J Xi L Gooding W Ferris RL Chemokine receptors 6 and 7 identify a metastatic expression pattern in squamous cell carcinoma of the head and neck Adv Otorhinolaryngol 2005 62 121 133 15608423 10.1159/000082501

[B19] Letsch A Keilholz U Schadendorf D Assfalg G Asemissen AM Thiel E Scheibenbogen C Functional CCR9 expression is associated with small intestinal metastasis J Invest Dermatol 2004 122 685 690 10.1111/j.0022-202X.2004.22315.x 15086554

[B20] Ghadjar P Coupland SE Na IK Noutsias M Letsch A Stroux A Bauer S Buhr HJ Thiel E Scheibenbogen C Keilholz U Chemokine receptor CCR6 expression level and liver metastases in colorectal cancer J Clin Oncol 2006 24 1910 1916 10.1200/JCO.2005.04.1822 16622267

[B21] Ghadjar P Loddenkemper C Coupland SE Stroux A Noutsias M Thiel E Christoph F Miller K Scheibenbogen C Keilholz U Chemokine receptor CCR6 expression level and aggressiveness of prostate cancer J Cancer Res Clin Oncol 2008 134 1181 1189 10.1007/s00432-008-0403-5 18465142 PMC12161728

[B22] Liu Z Fusi A Klopocki E Schmittel A Tinhofer I Nonnenmacher A Keilholz U Negative magnetic enrichment by immunomagnetic nanobeads for unbiased characterization of circulating tumor cells from peripheral blood of cancer patients J Transl Med 2011 9 70 10.1186/1479-5876-9-70 21595914 PMC3119001

[B23] Fusi A Reichelt U Busse A Ochsenreither S Rietz A Maisel M Keilholz U Expression of the Stem Cell Markers Nestin and CD133 on Circulating Melanoma Cells J Invest Dermatol 2011 131 487 94 10.1038/jid.2010.285 20882037

[B24] Hristozova T Konschak R Stromberger C Fusi A Liu Z Weichert W Stenzinger A Budach V Keilholz U Tinhofer I The presence of circulating tumor cells (CTCs) correlates with lymph node metastasis in nonresectable squamous cell carcinoma of the head and neck region (SCCHN) Ann Oncol 2011 22 1878 85 10.1093/annonc/mdr130 21525401

[B25] Pituch-Noworolska A Drabik G Szatanek R Białas M Kołodziejczyk P Szczepanik A Stachura J Zembala M Immunophenotype of isolated tumor cells in the blood, bone marrow and lymph nodes of patients with gastric cancer Pol J Pathol 2007 58 93 97 17715675

[B26] Reckamp KL Figlin RA Burdick MD Dubinett SM Elashoff RM Strieter RM CXCR4 expression on circulating pan-cytokeratin positive cells is associated with survival in patients with advanced non-small cell lung cancer BMC Cancer 2009 9 213 10.1186/1471-2407-9-213 19563666 PMC2708193

[B27] Pan J Mestas J Burdick MD Phillips RJ Thomas GV Reckamp K Belperio JA Strieter RM Stromal derived factor-I (SDF-I/CXCL12) and CXCR4 in renal cell carcinoma metastasis Mol Cancer 2006 5 56 10.1186/1476-4598-5-56 17083723 PMC1636662

[B28] Schimanski CC Schwald S Simiantonaki N Jayasinghe C Gönner U Wilsberg V Junginger T Berger MR Galle PR Moehler M Effect of chemokine receptors CXCR4 and CCR7 on the metastatic behaviour of human colorectal cancer Clin Cancer Res 2005 11 1743 50 10.1158/1078-0432.CCR-04-1195 15755995

[B29] Cabioglu N Yazici MS Arun B Broglio KR Hortobagyi GN Price JE Sahin A CCR7 and CXCR4 as novel biomarkers predicting axillary lymph node metastasis in T1 breast cancer Clin Cancer Res 2005 11 5686 93 10.1158/1078-0432.CCR-05-0014 16115904

[B30] Na IK Scheibenbogen C Adam C Stroux A Ghadjar P Thiel E Keilholz U Coupland SE Nuclear expression of CXCR4 in tumor cells of non-small cell lung cancer is correlated with lymph node metastasis Hum Pathol 2008 39 1751 5 10.1016/j.humpath.2008.04.017 18701133

[B31] Balkwill F The significance of cancer cell expression of the chemokine receptor CXCR4 Semin Cancer Biol 2004 14 171 9 10.1016/j.semcancer.2003.10.003 15246052

[B32] Schioppa T Uranchimeg B Saccani A Biswas SK Doni A Rapisarda A Bernasconi S Saccani S Nebuloni M Vago L Mantovani A Melillo G Regulation of the chemokine receptor CXCR4 by hypoxia J Exp Med 2003 198 1391 1402 10.1084/jem.20030267 14597738 PMC2194248

[B33] Yasuoka H Kodama R Hirokawa M Takamura Y Miyauchi A Sanke T Nakamura Y CXCR4 expression in papillary thyroid carcinoma: induction by nitric oxide and correlation with lymph node metastasis BMC Cancer 2008 8 274 10.1186/1471-2407-8-274 18826577 PMC2572635

[B34] Kim MY Oskarsson T Acharyya S Tumor Self-Seeding by Circulating Cancer Cells Cell 2009 139 1315 1326 10.1016/j.cell.2009.11.025 20064377 PMC2810531

[B35] Raynaud CM Mercier O Dartevelle P Commo F Olaussen KA de Montpreville V André F Sabatier L Soria JC Expression of chemokine receptor CCR6 as a molecular determinant of adrenal metastatic relapse in patients with primary lung cancer Clin Lung Cancer 2010 11 187 191 10.3816/CLC.2010.n.024 20439195

[B36] Rubie C Oliveira V Kempf K Wagner M Tilton B Rau B Kruse B Konig J Schilling M Involvement of chemokine receptor CCR6 in colorectal cancer metastasis Tumor Biol 2006 27 166 174 10.1159/000092777 16641550

[B37] Cabioglu N Sahin AA Morandi P Meric-Bernstam F Islam R Lin HY Bucana CD Gonzalez-Angulo AM Hortobagyi GN Cristofanilli M Chemokine receptors in advanced breast cancer: differential expression in metastatic disease sites with diagnostic and therapeutic implications Ann Oncol 2009 20 1013 1019 10.1093/annonc/mdn740 19237480 PMC4318926

[B38] Kaplan RN Riba RD Zacharoulis S Bramley AH Vincent L Costa C MacDonald DD Jin DK Shido K Kerns SA Zhu Z Hicklin D VEGFR1-positive haematopoietic bone marrow progenitors initiate the pre-metastatic niche Nature 2005 438 820 827 10.1038/nature04186 16341007 PMC2945882

[B39] Guleng B Tateishi K Ohta M Kanai F Jazag A Ijichi H Tanaka Y Washida M Morikane K Fukushima Y Yamori T Tsuruo T Blockade of the stromal cell-derived factor-1/CXCR4 axis attenuates in vivo tumor growth by inhibiting angiogenesis in a vascular endothelial growth factor-independent manner Cancer Res 2005 65 5864 5871 10.1158/0008-5472.CAN-04-3833 15994964

[B40] Rubin JB Kung AL Klein RS Chan JA Sun Y Schmidt K Kieran MW Luster AD Segal RA A small-molecule antagonist of CXCR4 inhibits intracranial growth of primary brain tumors Proc Natl Acad Sci USA 2003 100 13513 13518 10.1073/pnas.2235846100 14595012 PMC263845

[B41] Yasumoto K Koizumi K Kawashima A Saitoh Y Arita Y Shinohara K Minami T Nakayama T Sakurai H Takahashi Y Yoshie O Saiki I Role of the CXCL12/CXCR4 axis in peritoneal carcinomatosis of gastric cancer Cancer Res 2006 66 2181 2187 10.1158/0008-5472.CAN-05-3393 16489019

[B42] Richert MM Vaidya KS Mills CN Wong D Korz W Hurst DR Welch DR Inhibition of CXCR4 by CTCE-9908 inhibits breast cancer metastasis to lung and bone Oncol Rep 2009 21 761 767 19212637 PMC13435714

[B43] Huang EH Singh B Cristofanilli M Gelovani J Wei C Vincent L Cook KR Lucci A A CXCR4 antagonist CTCE-9908 inhibits primary tumor growth and metastasis of breast cancer J Surg Res 2009 155 231 236 10.1016/j.jss.2008.06.044 19482312

[B44] Hotte SJ Hirte HW Moretto P Iacobucci A Wong D Korz W Miller WH Final results of a phase I/II study of CTCE-9908, a novel anticancer agent that inhibits CXCR4, in patients with advanced solid cancers [abstract 405] Eur J Cancer Suppl 2008 6 127 127

